# PQ and Harmonic Assessment Issues on Low-Cost Smart Metering Platforms: A Case Study

**DOI:** 10.3390/s20216361

**Published:** 2020-11-07

**Authors:** Giovanni Artale, Giuseppe Caravello, Antonio Cataliotti, Valentina Cosentino, Dario Di Cara, Nunzio Dipaola, Salvatore Guaiana, Nicola Panzavecchia, Marilena G. Sambataro, Giovanni Tinè

**Affiliations:** 1 Department of Engineering, Università degli Studi di Palermo, 90128 Palermo, Italy; giovanni.artale@unipa.it (G.A.); giuseppe.caravello02@unipa.it (G.C.); antonio.cataliotti@unipa.it (A.C.); 2 Institute of Marine Engineering (INM), National Research Council (CNR), 90146 Palermo, Italy; guaiana.salvatore@inwind.it (S.G.); nicola.panzavecchia@cnr.it (N.P.); giovanni.tine@cnr.it (G.T.); 3 STMicroelectronics S.r.l., 95121 Catania, Italy; nunzio.dipaola@st.com (N.D.); marilena.sambataro@st.com (M.G.S.)

**Keywords:** power quality, smart meter, voltage and current sensing, harmonic analysis

## Abstract

This paper presents a feasibility study on how to implement power quality (PQ) metrics in a low-cost smart metering platform. The study is aimed at verifying the possibility of implementing PQ monitoring in distribution networks without replacing existing smart metering devices or adding new modules for PQ measurements, thus zeroing the installation costs. To this aim, an electronic board, currently used for remote energy metering, was chosen as a case study, specifically the STCOMET platform. Starting from the specifications of this device, the possibility of implementing power quality metrics is investigated in order to verify if compliance with standard requirements for PQ instruments can be obtained. Issues related to device features constraints are discussed; possible solutions and correction algorithms are presented and experimentally verified for different PQ metrics with a particular focus on harmonic analysis. The feasibility study takes into account both the use of on-board voltage and current transducers for low voltage applications and also the impact of external instrument transformers on measurement results.

## 1. Introduction

Nowadays smart metering devices have widespread use in many countries around the world to enable automatic meter reading (AMR) and other smart grid functionalities. Typical features include the measurement of different quantities, such as energy consumptions, supply voltages, frequency, absorbed currents and powers, as well as communication functionalities for enabling meter interaction with the grid and information exchange between customers and utilities. In this framework, several studies and proposals can be found in literature concerning both AMR, enhanced smart grid applications, and related measurement and communication issues. For example, in [1] and [2] main smart metering and communications technologies are surveyed, in the viewpoint of their integration in smart grid and industrial contexts; main features of metering and communication equipment and methods are analyzed, putting in evidence the advantages and open issues concerning advanced measurement and communication features. In [3], an overview is given of the enabling technologies and standards for communication, sensing, and monitoring purposes, also from the viewpoint of the full integration of distributed generation and storage systems at the customer level. In [4], a study is made on the possible integration of AMR with further sensing and IoT functionalities. In [5,6], some power line communication solutions for AMR applications are presented; refs. [7,8] wireless communication solutions for smart grid applications are addressed instead. Security issues and a possible privacy-preserving solution based on blockchain technology are investigated in [9]. The problem of real-time monitoring of low voltage distribution grids with distributed energy resources is addressed in [10,11], where proposed Information and Communication Technologies (ICT) architectures can integrate metering equipment at both users, prosumers, and distribution system operator level, to enable energy management and real-time grid monitoring. In [12], a metering and communication solution is proposed for the remote monitoring and control of distributed energy resources connected to low-voltage grids.

At the grid level, measurement instruments, such as power quality analyzers (PQAs) or phasor measurement units (PMUs), can be installed in different grid nodes (typically in transmission and distribution substations), for grid monitoring and management purposes, including PQ assessment [13]. This allows for the achievement of some data about the occurrence of PQ disturbances. In this framework, from the viewpoint of metering features, measurement reliability, and data availability, several issues have been investigated in the literature concerning this kind of instrumentation. For example, in [14] a solution is proposed to enhance distributed PMU availability during wired network failures. In [15,16], PMU new solutions are presented, aimed at reaching a good tradeoff between low cost and proper accuracy requirements. In [17], a study is carried out concerning the accuracy of signal processing algorithms for PMU measurements in the presence of disturbances in power system operating conditions. In [18], an enhanced solution for distribution system state estimation is presented, based on PMUs measurements and the cloud-based Internet of Things (IoT) paradigm. In [19,20,21], PQAs exploitation for power flows analysis at the distribution level is investigated, proposing properly distributed measurement systems and related power flows estimation algorithms able to also deal with partial data availability. In [22], PQ meters are integrated into a heterogeneous communication infrastructure to allow for the analysis of voltage dips in distribution networks.

However, more complete information about PQ levels across the grid could be reached by also including in the distributed measurement infrastructure PQ meters at the user level. For example, this could allow the implementation of billing strategies based not only on energy consumption but also on supply quality. In this framework, in [23] the study of a cost-effective microcontroller-based power quality meter is presented, which can be exploited at the user level for real-time monitoring of some PQ parameters. In [24], a prototype of an electronic power meter is developed, to integrate the measurement of typical power quality parameters and additional power terms for load characterization. In [25], a smart sensor network is proposed for non-intrusive load and power quality monitoring in both residential and industrial contexts. Among PQ disturbances, harmonic distortion is of great importance, especially in distribution grids, where the increasing presence of non-linear loads and power electronics in distributed generation and storage systems can strongly deteriorate the quality of supplied power and several electronic appliances and loads (at the home, commercial, and industrial level) are very sensitive to supply quality, especially in terms of harmonic distortion [26].

To enable diffused PQ measurements, it is important to introduce new technologies that can provide both end-users and distribution system operators with suitable information about the quality of the power supply at reasonable costs. In this framework, some studies and proposals can be found in research papers concerning the use of new energy meters, specifically developed for PQ measurement applications [27,28,29,30,31,32,33]. For example, in [27,28], an open-source energy and power quality meter is developed for low voltage applications at the user level. Further solutions for PQ measurements have been proposed in [29,30,31], which are based on microcontrollers and Raspberry-Pi. In [32], a multipurpose platform is developed for power system monitoring and analysis, which combines several measurement functionalities, i.e., power quality (PQ) analyzer, PMU, event logger, etc. A solution for a home area network application is proposed in [33], where the meter unit can be installed even on single loads and measured data are transmitted through a Radio-Frequency (RF) link to a wireless terminal unit, which works as a data logger and as a human–machine interface. In [34], a prototype is presented of a device and related metering algorithms for PQ monitoring, based on the Arduino NANO V3 ATmega328 microprocessor; the proposed device is integrated into a system for power supply reliability and power quality monitoring at both sides of switching devices.

It should be noticed that, in the viewpoint of widespread diffusion of PQ measurements at the user level, the installation of sophisticated and expensive instrumentation is not economically feasible. On the other hand, low-cost devices have been proposed to implement PQ measurement tasks; in this case, such additional instrumentation should be installed alongside the currently employed energy meters or new integrated platforms should replace those already used in distribution networks. In [35], the authors have investigated an alternative approach to the problem, i.e., the possibility of implementing PQ metrics on existing smart metering platforms; thus, exploiting current installed instrumentation and hardware architectures, by only modifying firmware and implemented metrics. This allows for the zeroing of the installation costs. The integration of PQ metrics into existing smart meters could open new market opportunities for both energy traders and manufacturers; moreover, this can allow users to easily monitor the quality of supplied power, without the need to handle different metering devices.

PQ phenomena measurements and limits are covered by specific standards [36,37,38,39,40]. The main reference is the IEC 61000-4-30, which defines the methods for PQ parameter measurement and the related accuracy requirements; as regards, harmonics (and interharmonics) measurements, it refers to IEC 61000-4-7. The instrument’s compliance with such standards is crucial to enable their use for monitoring or even billing purposes. In more detail, IEC 61000-4-30 defines two classes of measurements, i.e., classes A and S (correspondent to classes I and II of IEC 61000-4-7, respectively). Class A instruments are meant to be used for contractual applications or compliance verification with allowed disturbances limits; class S instruments are allowed for statistical applications such as surveys or power quality assessment and they obviously entail processing requirements lower than those of class A.

When dealing with PQ and harmonics measurements, another important issue to be considered is the influence of measurement transducers on the metrological accuracy of the whole measurement chain (i.e., transducers plus instrument) [41,42,43,44,45,46,47]. In fact, it is known that transducers can introduce relevant uncertainty contributions, especially when measured signals are not purely sinusoidal, because of transducer nonlinearities, frequency response, and bandwidth. In this viewpoint, PQ standards do not cover the presence of measurement transducers external to the instrument, and accuracy requirements do not include the transducers contribution. This problem is considered in the technical report IEC TR 61869-103 [38], which specifically deals with the use of instrument transformers (both inductive and electronic) for power quality measurements.

Starting from the aforesaid standard requirements for PQ and harmonic measurements, this paper investigates the issues related to the integration of PQ metrics on commercial platforms for smart metering. The device used as a case study is the STCOMET by STMicroelectronics [48], which is currently used in different countries for energy meter deployment. In detail, this paper is an extension of a previous author’s work [35], where a first implementation of some PQ metrics was presented. STCOMET already implements some PQ parameter metrics, such as frequency, RMS, dips, and swells. In [35], the compliance of on-board metrics with IEC 61000-4-30 was evaluated and some correction metrics were implemented and experimentally tested. In this way, the STCOMET measurement performances of the above-mentioned PQ parameters were found compatible with the accuracy class S requirements (in some cases even class A requirements were met). This paper investigates the possibility of implementing further metrics for harmonics measurement. Taking into account the constraints due to STCOMET technical specifications, a harmonic analysis algorithm was designed and implemented. Experimental tests were performed using the reference calibrator Fluke 6100A [49], in order to evaluate the compatibility between measurement errors and standard limits, according to IEC 61000-4-7. Furthermore, the impact of external measurement transducers on the whole measurement system accuracy is also analyzed; harmonics amplitude and phase errors were evaluated with and without external current transducers, to distinguish their contribution to measurement accuracy.

The paper is organized as follows. In Section 2, STCOMET specifications are analyzed from the viewpoint of obtaining measurement compliance with standard prescriptions, highlighting the main measurement issues to be considered for each accuracy class. Section 3 describes the implementation of the different PQ metric on STCOMET, the required correction algorithms, with a special focus on harmonic analysis. Section 4 reports the experimental characterization results for voltage and current measurements, both in sinusoidal and distorted conditions; tests were carried out both with and without an external current transformer, to evaluate its influence on the final measurement accuracy.

## 2. STCOMET Specifications and Standards Requirements

**Figure 1:**
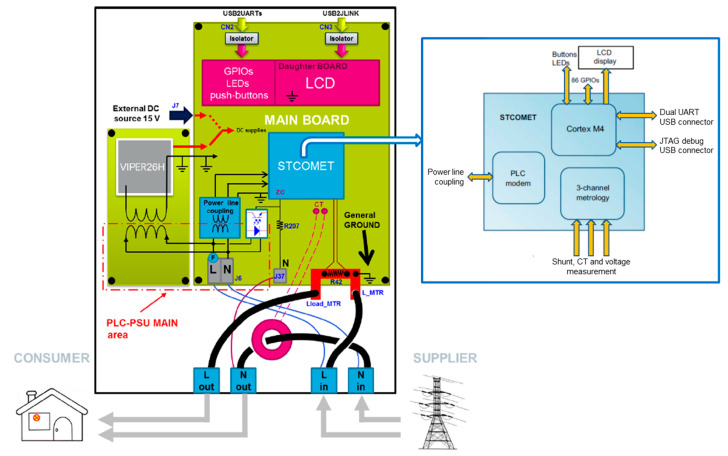
EVLKSTCOMET10-1 functional block diagram.

The evaluation kit for the STCOMET platform, named EVLKSTCOMET10-1 [48], was used as a case study to demonstrate the feasibility of the PQ measurement capability of an electronic board currently used for remote energy metering. EVLKSTCOMET10-1 includes a Cortex ™ -M4 microcontroller, a metrology section, and a power line communication (PLC) transceiver in a single device. The embedded PLC coupling circuit allows the STCOMET device to transmit and receive communication signals on the low voltage mains. More in detail, single carriers or OFDM (orthogonal frequency-division multiplexing) multi-carrier signals up to 500 kHz can be selected, enabling automatic meter reading (AMR) applications. The device functional block diagram is shown in Figure 1.

**Figure 2:**
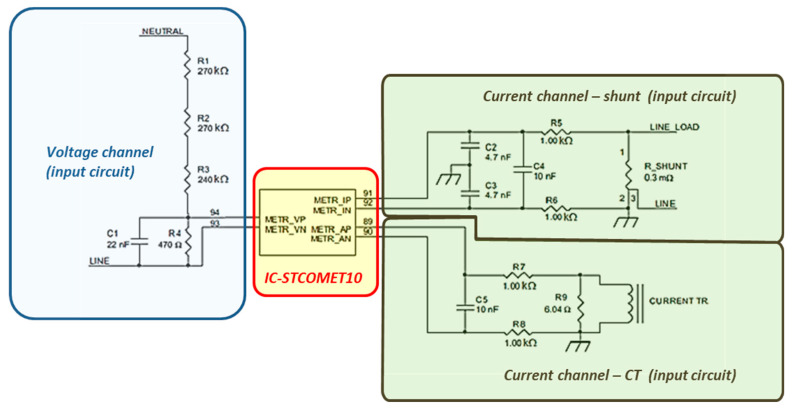
EVLKSTCOMET10-1 metrology reference application schematics.

As regards the measurement transducers, a resistive divider is used as a voltage sensor (Figure 2), while a shunt and a current transformer are available for current measurements (the shunt was used in experimental tests).

The metrology section includes a three-channel analog front end (AFE) with 24-bit sigma-delta converters and a dedicated digital signal processor (DSP). Available memories are: 640 kB or 1 MB of embedded Flash; 128 kB of embedded SRAM (static RAM); 8 kB of embedded shared RAM. EVLKSTCOMET10-1 metrological specifications are summarized in Table 1.

As mentioned in the introduction, IEC 61000-4-30 and IEC 61000-4-7 are the reference standards for PQ and harmonic measurements: IEC 61000-4-30 covers PQ measurement methods [36], referring to IEC 61000-4-7 for harmonics measurements [37]. Among the PQ parameters covered by the aforesaid standards, STCOMET base firmware already embeds metrics for power system frequency, voltage and current rms, and dips and swells. For those metrics, compatibility with standard requirements was analyzed in the paper, eventually suggesting correcting algorithms. The harmonic measurement metric is not available in the original firmware, thus it was developed, implemented, and tested.

According to IEC 61000-4-30, two possible classes are defined for measuring instruments: named A and S (corresponding to class I and II of IEC 61000-4-7, respectively). Class A is used when precise measurements are required (for example, in the case of billing applications that may require the resolution of disputes, detection of possible fraud, attribution of economical penalties for exceeding the standard limits, etc.) and class S is used for statistical applications (such as investigations of the origin of power quality disturbances, even with a limited subset of parameters PQ). For both classes, the base time interval (t.i.) for many measurements (voltage and current rms, harmonics, interharmonics, and unbalance) is 10/12 cycles for 50/60 Hz systems (i.e., 200 ms in the 50 Hz case). The 10/12 cycle values are then aggregated over three additional time intervals: 150/180 cycles, 10 min, and two hours.

**Figure 3:**
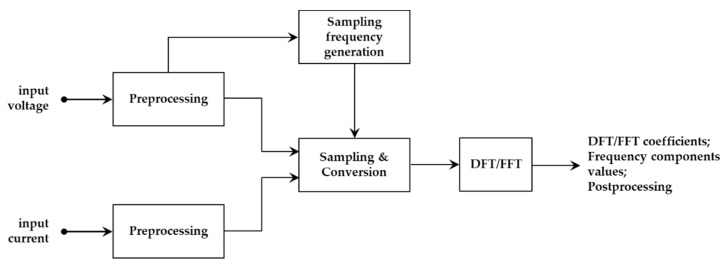
General structure of the IEC 61000-4-7 instrument.

As regards harmonic measurements, the IEC 61000-4-7 standard defines measurement requirements for harmonic distortion assessment both in terms of single harmonic component amplitudes and global parameters, such as the total harmonic distortion (THD) factor. The standard also provides indications regarding the general structure of measurement instruments, which is preferably based on the use of the discrete Fourier transform (DFT) and implemented by means of the fast Fourier transform (FFT) algorithm for shorter computation time (see Figure 3).

According to IEC 61000-4-7, the base time interval window must be synchronized to the fundamental power system frequency; the time between the leading edges of the first and the (*N + 1*)th sampling pulses (where *N* is the number of samples acquired in the observation window) shall be equal to the duration of the specified number of cycles, with a maximum allowable error of ±0.03% for class A instruments. Phase-locked loop or other synchronization systems are needed to properly generate the sampling frequency, in order to meet the aforesaid synchronization requirements. Hanning weighting is allowed only in the case of the loss of synchronization, even if in such occurrence the data shall be flagged and not used for the purpose of determining compliance with standard PQ limits.

On STCOMET, the input signal period is obtained by means of a zero-crossing method. Frequency is obtained from the period measurement. Using this method, it is necessary to filter harmonics and inter-harmonics, in order to minimize the effects of multiple zero crossings. This is in accordance with IEC 61000-4-30, which requires attenuating harmonics and inter-harmonics before measuring frequency. The STCOMET DSP calculates the period using the zero-crossing signal (ZRC) of the fundamental harmonic voltage, which is measured through a low-pass filter connected at the voltage channel. The resolution of the zero-crossing signal is 8 μs (given by the internal clock at 125 MHz); period measurement is updated every eight cycles of the input signal.

Even if the period is continuously measured, STCOMET sampling frequency cannot be synchronized to input signal frequency. In fact, as typically happens for many low-cost commercial platforms, STCOMET has a fixed sampling frequency. Thus, the synchronous sampling condition can be obtained only by properly choosing the number of acquired samples (*N*) in order to acquire an integer number of cycles of the input signal (10/12 cycles for the base time interval for power quality and harmonic measurements). In more detail, the STCOMET board has a fixed frequency sampling equal to *fs* = 7812.5 Hz (see Table 1), corresponding to a sampling period *Ts* = 128 µs. Considering a power system frequency of 50 Hz and the 10 cycles observation window *Tw* = 200 ms, the number of acquired samples is:

*N = Tw* × *fs* = 1562.5 ≈ 1563 (1)

FFT spectral resolution is:

Δ*F = fs/N* = 4.9984 Hz (2)

The synchronization error, corresponding to 0.5 samples, is of E = 0.5 × *Ts* = 64 µs, which in percentage terms can be calculated as follows:

*e% = (E/Tw*) × 100 = 0.032% > 0.03% (class A limit) (3)

Therefore, the instrument based on STCOMET cannot be classified in class A; thus, in the performed feasibility study class S requirements have been considered as a target for the implementation and verification of PQ and harmonics measurement metrics.

**Figure 4:**
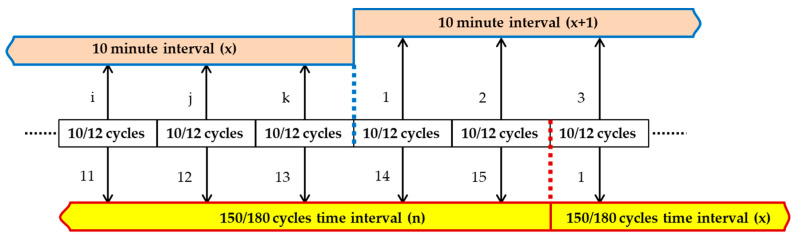
Aggregation intervals for Class S: parameters for which gaps are not permitted.

**Figure 5:**
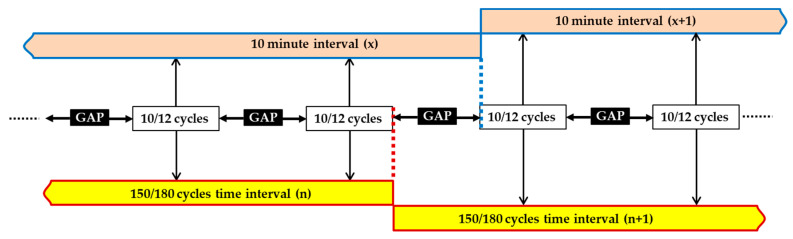
Aggregation intervals for class S: parameters for which gaps are permitted.

As regards the data aggregation over additional intervals, class S requirements are depicted in Figure 4 and Figure 5. In more detail, the data for the 150/180-cycles (i.e., 3 s) time interval shall be aggregated from fifteen contiguous 10/12-cycles (200 ms) base time intervals (see Figure 4). The aggregated data are calculated as the square root of the arithmetic mean of the squared input values measured in the base time intervals. For example, this is the case of RMS measurements. On the other hand, gaps are permitted for harmonics, inter-harmonics, mains signaling voltage, and unbalance (see Figure 5); in this case, a minimum of three 10/12-cycles values shall be used every 150/180-cycle time interval, i.e., at least one 10/12-cycles value shall be used every 50/60 cycles (1 s). This allows for the exploiting of the permitted gaps to reduce both the number of data to be aggregated (a minimum of one 10/12 cycles t.i. of every 50/60 cycles, for a total of three to mediate) and the computational time requirements. Slightly different aggregation intervals are defined in [38]; for example, 1 min aggregation intervals are considered for the assessment of voltage variations. In this framework, in [50] some comparative analyses have been made on the application of 1 and 10 min aggregation t.i., showing how the different t.i. and the requirements to have 100% of the measured data in the permissible range can pose some issues for the event assessment sensitivity, i.e., a single 1 min value can cause a negative assessment of voltage variation.

IEC 61000-4-7 accuracy requirements are reported in Table 2 for class I and class II instruments; the maximum allowable errors refer to single-frequency and steady-state signals, in the operating frequency range and under rated operating conditions indicated by the manufacturer (temperature and/or humidity ranges, rated supply voltage, etc.). No specific accuracy requirements are given for phase angle measurement.

Another important aspect to be addressed when dealing with power quality and harmonic measurements is the impact of measurement transducers. In fact, it is known that they are a key component of the measurement chain, which has a great impact on the overall measurement accuracy. In IEC 61000-4-30 the effect of transducers is acknowledged but not addressed in detail and external transducers and their associated uncertainty is not included in the accuracy requirements. The nature of this problem is better addressed in IEC TR 61869-103 [38], which specifically deals with the use of instrument transformers (both inductive and electronic) for power quality measurements. As detailed in [38], to evaluate transducer influence on the overall uncertainty on the measurement of a given power quality parameters, it is necessary to simultaneously consider the behavior of the transducer for a given disturbance and the related measurement method. Typically, the nonlinearity of the transducer can determine a worsening of the measurement uncertainty when the input signal (voltage or current) is not sinusoidal. The transducer impact on the overall uncertainty can be quantified as an added uncertainty that combines with the uncertainty of the measuring system. In this viewpoint, harmonic measurements can be negatively affected by the transducer presence, because of its frequency response (and bandwidth).

In this framework, the issues related to the implementation of harmonic analysis on STCOMET are analyzed in Section 3.2 with the aim of investigating the possibility of developing an instrument for harmonic measurements compliant with IEC 61000-4-7 accuracy requirements (class II). For phase angles measurements, quality metering specifications of IEC TR 61869-103 have [38] been used for reference; according to quality metering requirements of [38], phase errors at harmonics should be below 1° for 1st and 2nd harmonic and below 5° for other harmonics.

## 3. PQ Metrics Implementation on STCOMET

### 3.1. On-Board Metrics Compliance Verification and Correction Algorithms

As mentioned in the previous section, STCOMET already implements the measurement of: power system frequency; voltage and current RMS; and voltage dips and swells. The comparison between the IEC 61000-4-30 requirements for class S instruments and the STCOMET on-board metrics is reported in Table 3. To meet IEC 61000-4-30 requirements for on-board metrics, some correction algorithms have been implemented, as described in [35]. In brief:

− For frequency measurement, the main difference between IEC 61000-4-30 requirements and STCOMET on-board metric is the measurement time (10 s vs. 8 μs, i.e., STCOMET measurements are updated every 8 μs); thus the measurement over 10 s has been obtained as the mean of the frequency values (updated at every 8 μs) over the time interval of 10 s.
− For voltage/current RMS measurement, the main difference between the IEC 61000-4-30 requirements and STCOMET on-board metric is the observation window in subsequent measurements. In fact, IEC 61000-4-30 requires that the measurements shall be obtained over the base time interval (e.g., 10 cycles at 50 Hz); subsequent time intervals shall be contiguous, and not overlapping. STCOMET calculates RMS over the aforesaid base time interval; however, the RMS measurement is updated every 128 μs, i.e., once a new signal sample is available from the analog to digital converter (ADC). Thus, in the correction algorithm, the measurement reading update has been modified in order to obtain one RMS reading every 10 cycles.
− Main on-board metrics modifications were made for swells, dips, and interruptions. In fact, for swells and dips, IEC 61000-4-30 requires the measurement of a pair of data, i.e., the maximum/residual/voltage (i.e., the maximum/minimum voltage value recorded during the swell/dip) and the event duration, which is the difference between start and end times of the swell/dip (i.e., the time when the voltage RMS rises above/falls below the swell/dip threshold, and the time when the voltage RMS equals or goes back below/above the threshold); in this case, the RMS shall be measured over half a cycle or one cycle (Urms(1/2) or Urms(1), respectively). For interruptions, only event duration shall be measured. The STCOMET on-board metric was very different from the IEC 61000-4-30 one. Thus, a new algorithm was implemented, based on the measurement of Urms(1), as summarized in Table 3.

The aforesaid metrics were experimentally validated in [35]. In the following, further results are reported for frequency and RMS measurements, in both sinusoidal and distorted conditions which confirm the class S requirements compliance.

As regards the metrics not already on-board on STCOMET, in this study, voltage and current harmonics measurements have been implemented and tested, as described in the next sections.

### 3.2. Harmonics Analysis Implementation on STCOMET

As already mentioned, the EVLKSTCOMET10-1 uses a zero-crossing method for measuring the signal period/frequency, therefore this value can be used for the synchronization of the observation window *Tw* of 10/12 cycles of the 50/60 Hz input signal, i.e., 200 ms. If the frequency is not 50 Hz, the window would be greater or less than 200 ms. In accordance with [37], DFT/FFT algorithms have been evaluated for harmonic analysis. DFT can be performed with any number of samples *N*, but it has a higher computational cost (*O(N^2^)*); on the other hand, the FFT has a much lower computational cost (*O(NlogN)*), however, it needs a number of samples equal to a power of two to be performed (i.e., *N* = 1024, 2048, etc). An analysis of the computational time required for the algorithm executions with STCOMET leads to choose FFT (DFT execution time was not compatible with timing requirements for harmonic analysis even considering gaps).

To use the FFT, the closest values for *N* should be 2048. On the other hand, the STCOMET has a fixed sampling frequency of 7812.5 Hz, then the acquisition of 2048 samples would lead to a not synchronous observation window (i.e., *Tw =* 2048 × 128 µs ≈ 262 ms ≠ 200 ms). Thus, a time-domain interpolation algorithm has been implemented to obtain *N* = 2048 samples over the time interval of 10 cycles.

**Figure 6:**
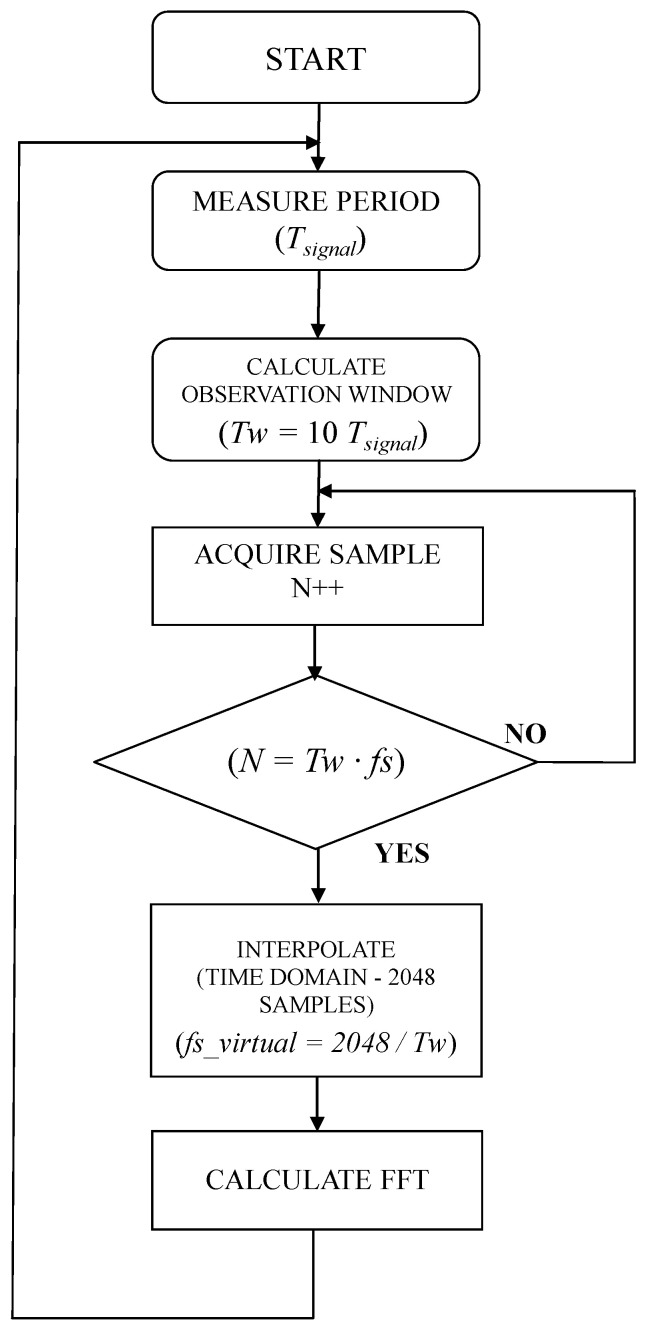
Flow operations performed by STCOMET.

Figure 6 shows a flow chart representing the operations of the algorithm implemented on STCOMET.

1. Starting from the measurement of the signal period *T_signal_* (carried out by STCOMET via zero-crossing, ZRC), an observation window equal to *Tw* = 10 *T_signal_* is set (for example, in the case of a 50 Hz signal, *T_signal_* = 20 ms, *Tw* = 200 ms).
2. The number of samples to be acquired and used for the algorithm is set: *N = Tw* × *fs*, where *fs* = 7812.5 Hz is the STCOMET sampling frequency (*M* = 1562.5 samples, rounding to the upper integer, i.e., 1563).
3. The time-domain interpolation of the acquired *N* samples is carried out to obtain 2048 samples for the FFT algorithm (a simple linear interpolation algorithm was used in the implementation, to minimize the computational cost). A virtual sampling frequency is determined, *fs_virtual*, which, in the same observation window *Tw*, would lead to the acquisition of the aforesaid 2048 samples:

*fs_virtual* = 2048/*Tw* (4)

In the case of *Tw* = 200 ms, *fs_virtual* = 10,240 Hz.

4. The FFT is calculated on the 2048 samples.

As regards the memory requirements, in the case of *f* = 50 Hz, *Tw* = 200 ms, and *fs* = 7812.5 Hz, the number of samples acquired is *M* = *Tw*/*fs* = 1563, so the number of bytes allocated in the STCOMET memory for samples storage is:

K = 1563 × 32 = 6.252 kB(5)

Considering that for the FFT calculation it is necessary to store 2048 samples (with both real and imaginary part), memory occupation is equal to 2048 × 32 × 2 = 16 kB. Furthermore, to allow the acquisition of samples of the subsequent observation window while performing the FFT calculation, it is necessary to instantiate a second register of 16 kB size. This was made in order to investigate the possibility of harmonic measurements without gaps between the observation windows. In summary, 32 kB are needed for samples storage and the FFT calculation algorithm; this requirement is compliant with STCOMET features, since the device has a RAM of 128 kB.

**Figure 7:**
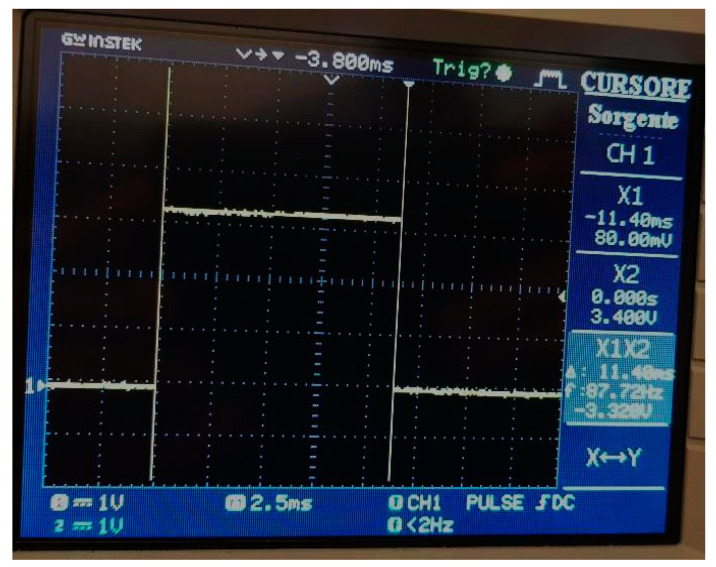
Time measured for the FFT calculation FFT.

In this way, whatever the input signal period is, the algorithm is able to virtually synchronize the observation window, in order to minimize the synchronization error. In addition, the “virtual re-sampling” of the signal allows for the calculation of the FFT on 2048 samples in the observation window of 10 signal periods, thus minimizing leakage and scallop loss errors. The adoption of a simple linear interpolation algorithm allows for the limiting of the computational cost of the harmonic calculation (FFT + interpolation). To demonstrate this, the FFT computation time was experimentally measured, both with and without the interpolation; as shown in Figure 7, the overall time needed for FFT with interpolation is 11.4 ms (a time of 9.6 ms was measured in the case of FFT calculation without interpolation). It should be noted that while executing the interpolation and FFT algorithms, the device continues acquiring the samples of the subsequent observation window, which are stored in memory for the next FFT calculation.

**Figure 8:**
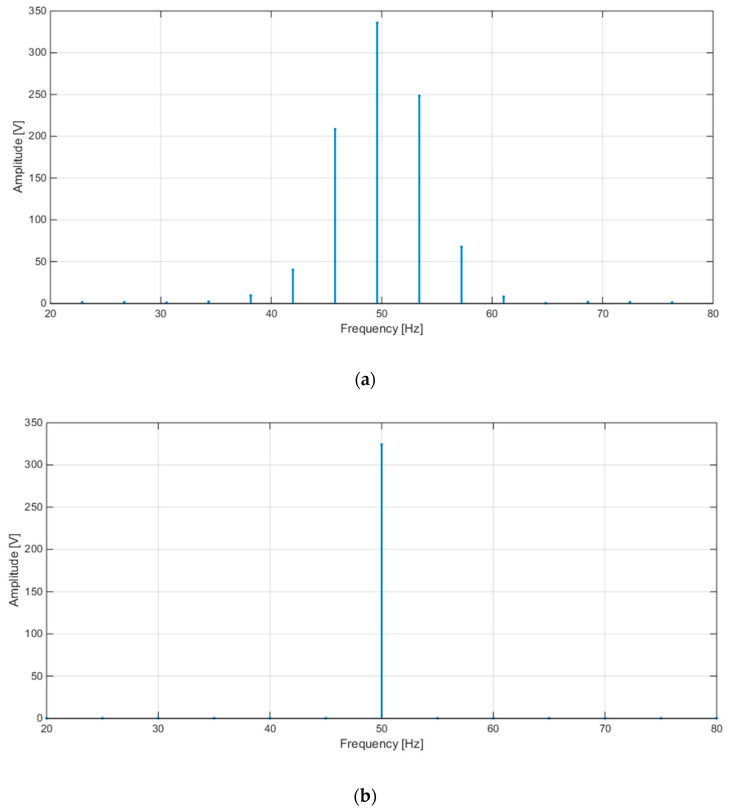
(**a**) FFT without interpolation; (**b**) FFT with interpolation.

Figure 8 shows the plots of the FFT calculated by STCOMET without interpolation (a) and with interpolation (b) (test with sinusoidal voltage, f = 50 Hz, Vrms = 230 V); as expected, the use of interpolation drastically reduces leakage and scallop loss errors.

## 4. Experimental Results

### 4.1. Test System

**Figure 9:**
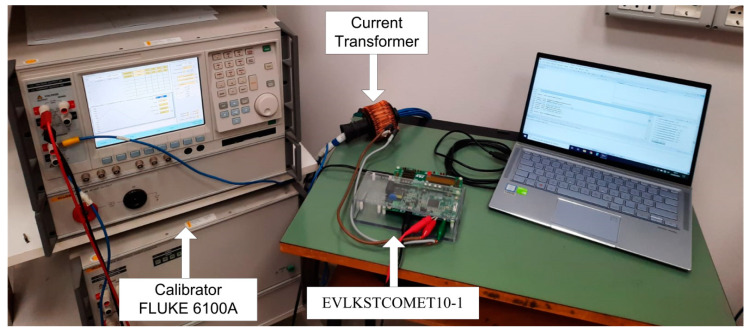
Test bench.

A measuring test bench was been set up as shown in Figure 9. To evaluate the PQ measurement performances, a calibrator was used as a reference for both voltage and current. The calibrator model used for experimental tests was the Fluke 6100A. Full accuracy specifications of the calibrator for PQ parameters are reported in the instrument manual [48]. Main accuracy specifications for the tests herein presented are the following: 50 ppm for frequency (setting the resolution to 0.1 Hz); up to ± (190 ppm of output + 33 mV) for fundamental and harmonic voltage amplitudes in the range from 70 to 1008 V for frequencies from 16 to 850 Hz and ± (524 ppm of output + 33 mV) for frequencies from 850 Hz to 6 kHz; up to ± (267 ppm of output + 720 μA) for fundamental and harmonic current amplitudes in the range from 2 to 21 A for frequencies from 16 to 850 Hz; up to 0.080° for current to voltage phase shift, for frequencies from 16 to 850 Hz and amplitudes from 0.5% to 40% of range. For the feasibility study presented in this work, the calibrator was assumed as a reference for the STCOMET characterization; for each measured quantity, the measurement system error was calculated as the difference between the value set on the calibrator and that measured with STCOMET.

Voltage measurements were directly acquired by STCOMET. To investigate the impact of external measurement transducers, current measurements were carried out with and without an external current transformer (CT); in both cases, the STCOMET current channel with internal shunt was used. The external current transformer is an open window CT used by Italian utilities to connect energy meters to low voltage distribution networks. The CT-rated data are summarized in Table 4. The window diameter is 3 cm. The EVLKSTCOMET10-1 has been connected to a PC via USB JTAG J-linkOB. STCOMET firmware was updated with both the corrected on-board metrics and the harmonic analysis algorithm using the IAR Embedded Workbench.

Tests were carried out reproducing different PQ phenomena (harmonics, dips, and swell interruptions) and for different frequencies of the test signal (in the range of 42.5 ÷ 57.5 Hz). Some results for dips, swells, and interruptions can be found in [35]. In the following sections, some results for frequency and voltage/current RMS and harmonics measurements are presented. For current measurements, both amplitudes and phase errors are shown.

### 4.2. Voltage Measurements

Tests have been made in both sinusoidal and distorted conditions.

Results obtained with the FFT algorithm are shown, together with RMS and frequency measurements; as already mentioned, errors between values generated by the calibrator and those measured by STCOMET are calculated and compared with IEC 61000-4-30 and IEC 61000-4-7 accuracy requirements. In more detail, calculated errors are listed in Table 5.

where:

- *f_FFT* e *V_FFT* are frequency and amplitude (in RMS) measured by STCOMET (fundamental and harmonics);
- *f_gen* e *V_gen* are frequency and amplitude (in RMS) generated by the calibrator (fundamental and harmonics).

**Figure 10:**
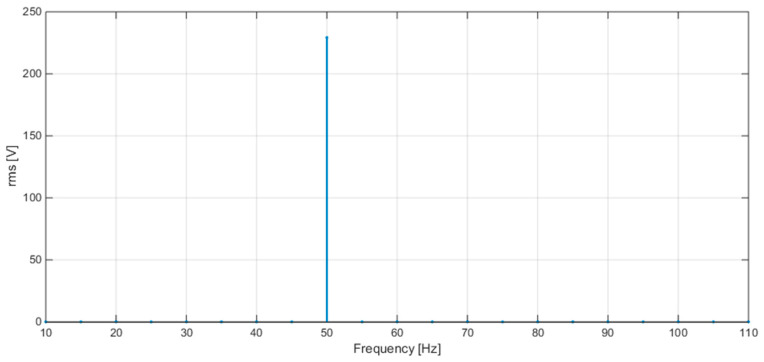
FFT plot values obtained with STCOMET using interpolation (V = 230 V, f = 50 Hz).

**Figure 11:**
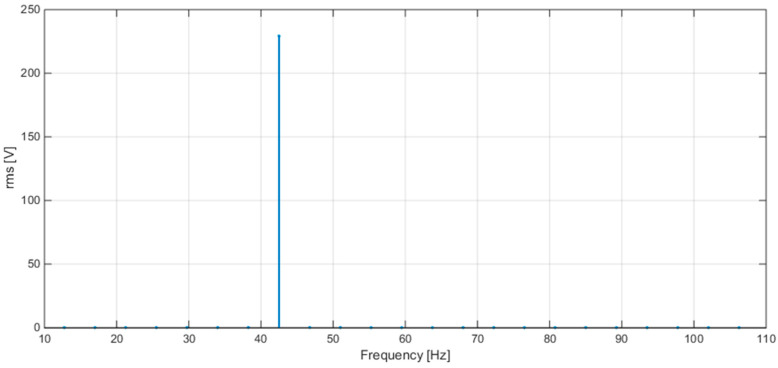
FFT plot values obtained with STCOMET using interpolation (V = 230 V, f = 42.5 Hz).

**Figure 12:**
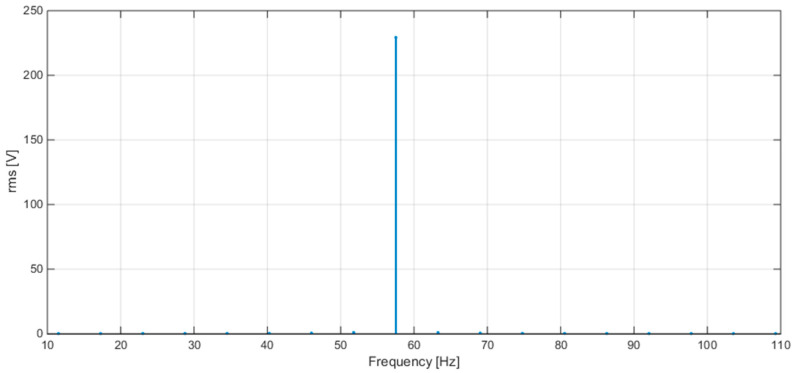
FFT plot values obtained with STCOMET using interpolation (V = 230 V, f = 57.5 Hz).

Some results of tests with sinusoidal voltage are reported in Table 6, for different frequency values (50, 42.5, and 57.5 Hz). Figure 10, Figure 11 and Figure 12 show the spectra obtained at the three frequencies 50, 42.5, and 57.5 Hz, respectively. The obtained errors are compatible with the limits set by IEC 61000-4-30 for class S instruments (equal to ±50 mHz and ±0.5% for frequency and RMS measurements, respectively).

Further tests were performed with distorted voltage signals, with 25 harmonics, choosing the harmonic amplitudes equal to the limit values for electrical networks reported in CEI EN 50160 [40] and shown in Table 7. The voltage of the fundamental harmonic generated by the calibrator was V1 = 230 V and the RMS voltage was V = 231.47 V. The tests were carried out for different frequency values.

In Table 8, the results of the first test performed at a fundamental frequency *f*_1_ = 50 Hz are reported. As can be seen, the measurement results are within the class S requirements (for frequency also class A requirements are met).

The measured errors for each harmonic are reported in Table 9. Comparing these values with that of the limits reported in Table 2, it is possible to observe that the measuring system is compatible with the class II requirements (in some cases measurement results comply also with class I limits).

- f_gen is the frequency generated by the calibrator;
- V_gen is the voltage generated by the calibrator;
- f_FFT is the frequency measured using the FFT algorithm;
- E is the absolute error between f_FFT and f_gen expressed in mHz;
- V_FFT is the measured voltage;
- Em is the error on the measured value calculated (see Table 2);
- En is the error on the nominal value (see Table 2).

The same test with 25 harmonics was repeated for different signal frequencies. Some results are reported in the following tables for 42.5 Hz (see Table 10 and Table 11) and 57.5 Hz (see Table 12 and Table 13). Even in these cases, measurement results are within the class S maximum allowable errors for RMS and frequency and they are compatible with the class II requirements for harmonic measurements (for frequency also class A requirements are met and in many cases errors on harmonics are within class I limits).

Further tests were carried out by changing the phase shift between harmonic and fundamental components of the voltage test signal. This allowed for the investigation of the influence of harmonic phase shift, which can negatively affect the measurement accuracy, especially in the presence of measurement transducers. Tests herein presented were performed with a distorted voltage with fundamental frequency *f*_1_ = 50 Hz, fundamental amplitude *V*_1_ = 230 V, and four harmonics, i.e., the II, III, VI, and XI order harmonics. Each harmonic has an amplitude equal to 10% of the fundamental. For each harmonic, phase shift with respect to the fundamental harmonic was changed from −180° to +180°, with steps of 30°. Table 14 and Table 15 show amplitude and phase errors in harmonic voltage measurements, respectively. It can be seen that the proposed measurement system complies with class I requirements.

### 4.3. Current Measurements

The tests made for voltage were repeated also for currents. As regards RMS and FFT measurements, the results obtained were similar to those obtained for voltage measurements.

The tests with harmonics at different phase-shifts were carried out with and without the external CT, in order to investigate not only the STCOMET behavior in current measurements but also the external CT influence on the measurement results. Tests herein presented were carried out at 50 Hz, with fundamental current amplitude *I*_1_ = 5A; II, III, VI, and XI order harmonics have been considered, with an amplitude equal to the 10% of fundamental current and variable phase shift from −180° to +180°, with steps of 30°.

Table 16 and Table 17 show amplitude and phase errors in harmonic current measurements, respectively, for the tests without the external CT. Also, in this case, the proposed measurement system complies with class I requirements.

The aforesaid tests were repeated with the external CT, whose secondary winding was connected to the on-board shunt (see test bench of Figure 9). To obtain the rated current (125 A), a 10-turns primary winding was set up. Each turn had a very large diameter compared to the CT window diameter. Moreover, the 10 loops of the winding were wound in a compact group, with a small section compared to the CT window area. In this way, the current generated by the calibrator was 12.5 A and it had the same magnetic effects as a 125 A equivalent current flowing in a single cable.

Table 18 and Table 19 show amplitude and phase errors in current measurements, respectively. It can be seen that the measured errors are higher in comparison to those of Table 16 and Table 17 because they are strongly influenced by the CT behavior. Moreover, they have a large variability with the harmonic phase shift with respect to fundamental. This behavior is in agreement with what was found in previous works on the CT metrological performances in distorted conditions [47].

## 5. Conclusions

The paper has verified the possibility to implement PQ metrics into smart meters used for remote energy metering purposes. The aim of this feasibility study was to demonstrate that PQ distributed monitoring can be performed in a distribution network without replacing already installed smart meters or adding new PQ modules. In fact, the proposed solution entails a firmware upgrade, which implements the power quality metrics using the available calculation capabilities. Moreover, using the metering infrastructure already installed and the power line communication capabilities of the smart meters, PQ measurements can be collected, and statistical analysis can be performed all over distribution networks.

The feasibility of PQ metrics implementation in smart meters was demonstrated by choosing the real case study of STCOMET, an electronic board widely used for remote energy metering purposes in different countries. Starting from an analysis of the electronic board specifications, it was verified that the fixed sampling frequency value results in a synchronization error outside class A requirements. Thus, class S instrument prescriptions were considered as a reference both for PQ metrics and for data aggregation over additional intervals. Different correction algorithms of on-board metrics were implemented in order to comply with standard requirements for each parameter to be measured, i.e., frequency, voltage, and current RMS; voltage dips and swells. As regards harmonic measurements, the considered board has no predefined metrics. Thus, different solutions were considered for harmonic analysis implementation. The analysis led to the choice of the FFT algorithm with interpolation. In this way, the signal is “virtually re-sampled” minimizing leakage and scallop loss errors. The adoption of a simple linear interpolation algorithm allows for the limiting of the computational cost of the harmonic calculation (FFT + interpolation), which is compatible with the board capabilities. Moreover, the calculation times were measured and found to be compatible with standard requirements, thus confirming the feasibility of the proposed solution. The compliance with accuracy standard requirements was then verified with experimental tests. Different distorted conditions were tested, varying the frequency of the signal and the harmonic content, in terms of both harmonic component amplitudes and phase shifts. Summarizing the results of all the tested cases, the study has shown that correction algorithms applied to on-board metrics allowed for the meeting of IEC 61000-4-30 requirements for class S instruments for the following measurement quantities: frequency; voltage and current, RMS; interruptions, voltage dips, and swells. Moreover, thanks to the developed harmonic measurement algorithm, the smart meter fulfills the class II requirements for harmonic measurements according to IEC 61000-4-7. In many cases, errors on harmonics were even found within class I limits. However, the compatibility with class S and class II requirements grants that smart meters enhanced with the developed firmware can be correctly used for statistical applications, such as surveys or power quality assessment all over an LV network. In this viewpoint, future work will be focused on using these new measurement capabilities in a distributed measurement architecture, developing and testing new algorithms for fault detection and fault diagnosis, extensive power quality surveys, and harmonic distortion assessment.

Finally, some tests were performed to study the effect on harmonic measurements of additional external transducers, specifically a current transformer used to couple energy meters to LV networks. As expected, larger errors were found which have a larger variability with harmonic phase shift with respect to fundamental. Future work will be focused on implementing on-board correction techniques of CT errors in distorted condition, thus reducing the total uncertainty associated with the whole measurement chain.

## Figures and Tables

**Table 1:** EVLKSTCOMET10-1 metrological main specifications.

Parameter	Value
Nominal input voltage	230 V
Nominal line current	5 A
Nominal input frequency	50/60 Hz
Sampling frequency (Fs)	7.8125 kHz
Bandwidth (–3 dB)	0–3.6 kHz
Voltage/current RMS accuracy	0.5%

**Table 2:** Maximum errors of voltage and current according to IEC 61000-4-7.

Class	Measurement	Conditions	Maximum Error
I	Voltage	$U_{m}\geq 1\%\;U_{nom}$ $U_{m}<1\%\;U_{nom}$	$\pm 5\%\;U_{m}$ $\pm 0.05\%\;U_{nom}$
	Current	$I_{m}\geq 3\%\;I_{nom}$ $I_{m}<3\%\;I_{nom}$	$\pm 5\%\;I_{m}$ $\pm 0.15\%\;I_{nom}$
	Power	$P_{m}\geq 150W$ $P_{m}<150W$	$\pm 1\%\;P_{m}$ ±1.5W
II	Voltage	$U_{m}\geq 3\%\;U_{nom}$ $U_{m}<3\%\;U_{nom}$	$\pm 5\%\;U_{m}$ $\pm 0.15\%\;U_{nom}$
	Current	$I_{m}\geq 10\%\;I_{nom}$ $I_{m}<10\%\;I_{nom}$	$\pm 5\%\;I_{m}$ $\pm 0.5\%\;I_{nom}$

**Table 3:** STCOMET on-board metrics vs. IEC 61000-4-30 requirements (class S) [33].

Parameter	IEC 61000-4-30			STCOMET Metric Main Features	Correction Algorithms
	Measurement Range	Time Interval (t.i.)	Maximum Error		
Frequency Period	42.5 ÷ 57.5 Hz (for 50 Hz syst.)	10 s (not overlapping)	− Number of integer cycles during the 10-s t.i. divided by their cumulative duration − Harmonics and interharmonics are attenuated	− Measurement from zero-crossing (voltage channel, with a low pass filter). − Frequency range between 32.55 and 81.38 Hz − Resolution of 8 μs − Period is calculated as the mean of the last eight measured periods	− Single frequency value is obtained as period reciprocal (on-board metric) − Frequency measurement is calculated as the mean of the single frequency values over 10 s
RMS	20 ÷ 120% Udin	200 ms (10 cycles for 50 Hz) no gaps	− The r.m.s. value includes, by definition, harmonics, interharmonics, etc. − Every 10/12-cycle interval shall be contiguous and not overlapping − Not used for dips, swells, voltage interruptions, and transients	− RMS measurement is obtained from voltage/current samples − Voltage/current are not filtered − Integration time is 10 cycles − RMS value is updated every 128 μs	− No modifications of on-board metric − Measurement reading is updated every 10 cycles, in order to have not overlapping t.i.
Dips Swells Interruptions	---	---	− The basic measurement shall be the RMS over a half cycle or full cycle (Urms(1/2) or Urms(1)) − Measured data are maximum/residual voltage (i.e., the max./min. voltage value during the event) and duration (i.e., the difference between start and end times of the event) − For interruptions only duration is measured	− The fundamental component of voltage (RMS) is compared to a 10-bit threshold − An internal time counter is incremented until the momentary voltage value is below/above the threshold − Maximum/residual voltage is not calculated − Interruptions measurement is not on-board	− (Urms(1)) has been implemented − Urms(1) is compared with the event threshold to obtain event start, end, and duration − An internal counter is incremented until Urms(1) value is below/above the threshold − Urms(1) measurement is used to store the residual/max. voltage
Harmonics	10 ÷ 100% of harmonic limits, up to 40th order	200 ms (gaps allowed) ± 0.03% max. synchr. error (optional)	− DFT/FFT − t.i. synchronized to the power system frequency − Rectangular window (Hanning in case of loss of synchronization	− Not on-board	− New algorithm (see Section 3.2)

**Table 4:** External current transformer rated data.

	CT
Transformer ratio	125/5 A/A
Standard burden	2.5 VA
Class	0.5
Rated frequency	50 Hz
Rated voltage	400 V

**Table 5:** Error definitions.

Parameter	Description
*E = |f_FFT − f_gen|*	Error, in mHz, on the frequency measurements both for fundamental and harmonics obtained via FFT
*e% = 100* × *|V_FFT − V_gen|/V_gen*	Percentage error on amplitude measurements obtained via FFT (fundamental and harmonics of amplitude greater than 3% of the rated voltage, *V_nom*, i.e., 230 V)
*e% = 100* × *|V_FFT − V_gen|/V_nom*	Percentage error on amplitude measurements obtained through FFT (harmonics of amplitude less than 3% of the nominal voltage *V_nom*)

**Table 6:** Voltage measurements in sinusoidal tests at different frequencies.

Calibrator Settings		STCOMET Measurements and Related Errors			
		Output FFT			
f_gen (Hz)	V_gen (V)	f1_FFT (Hz)	E (mHz)	V1_FFT (V)	e%
50.0	230	50.0000	0.0	229.26	0.32
42.5	230	42.5025	2.5	229.28	0.31
57.5	230	57.524	24	229.27	0.31

**Table 7:** CEI EN 50160—harmonic voltages amplitudes at the power supply terminals (% of the fundamental voltage).

Odd Harmonics				Even Harmonics	
Non-Multiple of 3		Multiple of 3			
Harmonic Order h	Relative Amplitude U_h_	Harmonic Order h	Relative Amplitude U_h_	Harmonic Order h	Relative Amplitude U_h_
5	6.0%	3	5.0%	2	2.0%
7	5.0%	9	1.5%	4	1.0%
11	3.5%	15	0.5%	6…24	0.5%
13	3.0%	21	0.5%		
17	2.0%				
19	1.5%				
23	1.5%				
25	1.5%				

**Table 8:** RMS and frequency measurement results. Distorted voltage signal with 25 harmonics, *f*_1_ = 50 Hz.

	Supply Values	Measured Value	Error	Maximum Error IEC 61000-4-30	Class A/S
RMS	231.47 (V)	230.70 (V)	0.33%	±0.5% $U_{din}$ ^1^	S
Frequency	50.0 (Hz)	50.0000 [Hz]	0.0 (mHz)	±10 mHz	A

^1^$U_{din}$ is the declared supply voltage.

**Table 9:** FFT results. Distorted voltage signal with 25 harmonics; *f*_1_ = 50 Hz.

	f_gen	V_gen	V_gen	f_FFT	E	V_FFT	Em	En	Class I	Class II	Class
	(Hz)	(V)	(%)	(Hz)	(mHz)	(V)	(%)	(%)	Limits	Limits	Limits
1	50	230	100%	50.0000	0.0	229.25	0.33%	0.32%	True	True	I
2	100	4.6	2.0%	100.000	0.0	4.586	0.30%	0.006%	True	True	I
3	150	11.5	5.0%	150.000	0.0	11.460	0.35%	0.017%	True	True	I
4	200	2.3	1.0%	200.000	0.0	2.289	0.48%	0.005%	True	True	I
5	250	13.8	6.0%	250.000	0.0	13.705	0.69%	0.041%	True	True	I
6	300	1.15	0.5%	300.000	0.0	1.139	0.98%	0.005%	True	True	I
7	350	11.5	5.0%	350.000	0.0	11.38	1.0%	0.051%	True	True	I
8	400	1.15	0.5%	400.000	0.0	1.136	1.3%	0.006%	True	True	I
9	450	3.45	1.5%	450.000	0.0	3.402	1.4%	0.021%	True	True	I
10	500	1.15	0.5%	500.000	0.0	1.130	1.8%	0.009%	True	True	I
11	550	8.05	3.5%	550.000	0.0	7.890	2.0%	0.070%	True	True	I
12	600	1.15	0.5%	600.000	0.0	1.124	2.2%	0.011%	True	True	I
13	650	6.9	3.0%	650.000	0.0	6.720	2.6%	0.079%	True	True	I
14	700	1.15	0.5%	700.000	0.0	1.115	3.1%	0.015%	True	True	I
15	750	1.15	0.5%	750.000	0.0	1.113	3.2%	0.016%	True	True	I
16	800	1.15	0.5%	800.000	0.0	1.106	3.8%	0.019%	True	True	I
17	850	4.6	2.0%	850.000	0.0	4.40	4.3%	0.086%	True	True	I
18	900	1.15	0.5%	900.000	0.0	1.096	4.7%	0.023%	True	True	I
19	950	3.45	1.5%	950.000	0.0	3.27	5.2%	0.079%	False	True	II
20	1000	1.15	0.5%	1000.00	0.0	1.083	5.8%	0.029%	True	True	I
21	1050	1.15	0.5%	1050.00	0.0	1.078	6.2%	0.031%	True	True	I
22	1100	1.15	0.5%	1100.00	0.0	1.070	7.0%	0.035%	True	True	I
23	1150	3.45	1.5%	1150.00	0.0	3.19	7.6%	0.11%	False	True	II
24	1200	1.15	0.5%	1200.00	0.0	1.058	8.0%	0.040%	True	True	I
25	1250	3.45	1.5%	1250.00	0.0	3.15	8.8%	0.13%	False	True	II

**Table 10:** RMS and frequency measurement results. Distorted voltage signal with 25 harmonics, *f*_1_ = 42.5 Hz.

	Supply Values	Measured Value	Error with Respect to the Calibrator	Maximum Error IEC 61000-4-30	Class A/S
RMS	231.47 (V)	230.66 (V)	0.35%	±0.5% $U_{din}$	S
Frequency	42.5 (Hz)	42.5025 (Hz)	2.5 mHz	±10 mHz	A

**Table 11:** FFT results. Distorted voltage signal with 25 harmonics, *f*_1_ = 42.5 Hz.

Harm	f_gen	V_gen	V_gen	f_FFT	E	V_FFT	Em	En	Class I	Class II	Class
	(Hz)	(V)	(%)	(Hz)	(mHz)	(V)	(%)	(%)	Limits	Limits	Limits
1	42.5	230	100%	42.5026	2.6	229.33	0.29%	0.289%	True	True	I
2	85	4.6	2.0%	85.0051	5.1	4.588	0.26%	0.005%	True	True	I
3	127.5	11.5	5.0%	127.508	7.7	11.462	0.33%	0.016%	True	True	I
4	170	2.3	1.0%	170.010	10	2.290	0.46%	0.005%	True	True	I
5	212.5	13.8	6.0%	212.513	13	13.726	0.53%	0.032%	True	True	I
6	255	1.15	0.5%	255.015	15	1.1435	0.56%	0.003%	True	True	I
7	297.5	11.5	5.0%	297.518	18	11.408	0.80%	0.040%	True	True	I
8	340	1.15	0.5%	340.020	20	1.1423	0.67%	0.003%	True	True	I
9	382.5	3.45	1.5%	382.523	23	3.415	1.0%	0.015%	True	True	I
10	425	1.15	0.5%	425.026	26	1.138	1.1%	0.005%	True	True	I
11	467.5	8.05	3.5%	467.528	28	7.93	1.5%	0.051%	True	True	I
12	510	1.15	0.5%	510.031	31	1.131	1.7%	0.008%	True	True	I
13	552.5	6.9	3.0%	552.533	33	6.76	2.0%	0.061%	True	True	I
14	595	1.15	0.5%	595.036	36	1.126	2.1%	0.010%	True	True	I
15	637.5	1.15	0.5%	637.538	38	1.122	2.4%	0.012%	True	True	I
16	680	1.15	0.5%	680.041	41	1.118	2.8%	0.014%	True	True	I
17	722.5	4.6	2.0%	722.543	43	4.47	2.9%	0.058%	True	True	I
18	765	1.15	0.5%	765.046	46	1.114	3.1%	0.016%	True	True	I
19	807.5	3.45	1.5%	807.548	48	3.32	3.7%	0.055%	True	True	I
20	850	1.15	0.5%	850.051	51	1.103	4.0%	0.020%	True	True	I
21	892.5	1.15	0.5%	892.554	54	1.101	4.3%	0.021%	True	True	I
22	935	1.15	0.5%	935.056	56	1.094	4.9%	0.024%	True	True	I
23	977.5	3.45	1.5%	977.559	59	3.27	5.2%	0.079%	False	True	II
24	1020	1.15	0.5%	1020.061	61	1.083	5.9%	0.029%	True	True	I
25	1063	3.45	1.5%	1062.564	64	3.23	6.4%	0.095%	False	True	II

**Table 12:** RMS and frequency measurement results. Distorted voltage signal with 25 harmonics, *f*_1_ = 57.5 Hz.

	Supply Values	Measured Value	Error with Respect to the Calibrator	Maximum Error IEC 61000-4-30	Class A/S
RMS	231.47 (V)	230.70 (V)	0.33%	±0.5% $U_{din}$	S
Frequency	57.5 (Hz)	57.4977 (Hz)	2.3 mHz	±10 mHz	A

**Table 13:** FFT results. Distorted voltage signal with 25 harmonics, *f*_1_ = 57.5 Hz.

Harm	f_gen	V_gen	V_gen	f_FFT	E	V_FFT	Em	En	Class I	Class II	Class
	(Hz)	(V)	(%)	(Hz)	(mHz)	(V)	(%)	(%)	Limits	Limits	Limits
1	57.5	230	100%	57.4977	2.3	229.31	0.30%	0.300%	True	True	I
2	115	4.6	2.0%	114.9954	4.6	4.585	0.32%	0.006%	True	True	I
3	172.5	11.5	5.0%	172.4931	6.9	11.454	0.40%	0.020%	True	True	I
4	230	2.3	1.0%	229.9908	9.2	2.288	0.52%	0.005%	True	True	I
5	287.5	13.8	6.0%	287.489	12	13.69	0.81%	0.049%	True	True	I
6	345	1.15	0.5%	344.986	14	1.139	0.93%	0.005%	True	True	I
7	402.5	11.5	5.0%	402.484	16	11.36	1.2%	0.062%	True	True	I
8	460	1.15	0.5%	459.982	18	1.132	1.6%	0.008%	True	True	I
9	517.5	3.45	1.5%	517.479	21	3.384	1.9%	0.029%	True	True	I
10	575	1.15	0.5%	574.977	23	1.124	2.2%	0.011%	True	True	I
11	632.5	8.05	3.5%	632.475	25	7.84	2.6%	0.091%	True	True	I
12	690	1.15	0.5%	689.972	28	1.116	3.0%	0.015%	True	True	I
13	747.5	6.9	3.0%	747.470	30	6.66	3.4%	0.10%	True	True	I
14	805	1.15	0.5%	804.968	32	1.10	4.1%	0.021%	True	True	I
15	862.5	1.15	0.5%	862.466	35	1.09	5.2%	0.026%	True	True	I
16	920	1.15	0.5%	919.963	37	1.09	5.1%	0.025%	True	True	I
17	977.5	4.6	2.0%	977.461	39	4.34	5.7%	0.11%	False	True	II
18	1035	1.15	0.5%	1034.959	41	1.07	6.5%	0.032%	True	True	I
19	1093	3.45	1.5%	1092.456	44	3.21	6.9%	0.10%	False	True	II
20	1150	1.15	0.5%	1149.954	46	1.06	7.8%	0.039%	True	True	I
21	1208	1.15	0.5%	1207.452	48	1.05	9.0%	0.045%	True	True	I
22	1265	1.15	0.5%	1264.949	51	1.04	9.3%	0.046%	True	True	I
23	1323	3.45	1.5%	1322.447	53	3.10	10%	0.15%	False	True	II
24	1380	1.15	0.5%	1379.945	55	1.03	10%	0.053%	True	True	I
25	1438	3.45	1.5%	1437.443	58	3.05	11%	0.17%	False	True	II

**Table 14:** Voltage module error for II, IV, VI, and XI order harmonics.

	Amplitude Error		
Harmonic Order	Min	Max	Average
II	0.013%	0.13%	0.071%
III	0.001%	0.12%	0.047%
VI	0.084%	0.12%	0.11%
XI	0.12%	0.32%	0.27%

**Table 15:** Voltage phase error for II, IV, VI, and XI order harmonics.

	Phase Error (deg)		
Harmonic Order	Min	Max	Average
II	0.089	0.15	0.12
III	0.028	0.088	0.043
VI	0.065	0.11	0.086
XI	0.089	0.14	0.12

**Table 16:** Current amplitude error for II, IV, VI, and XI order harmonics.

	Amplitude Error		
Harmonic Order	Min	Max	Average
II	0.087%	0.31%	0.21%
III	0.073%	0.53%	0.28%
VI	0.020%	0.5%	0.32%
XI	0.039%	1.12%	0.27%

**Table 17:** Current phase error for II, IV, VI, XI order harmonics.

	Phase Error (deg)		
Harmonic Order	Min	Max	Average
II	0.050	0.31	0.18
III	0.015	0.25	0.096
VI	0.001	0.13	0.062
XI	0.024	0.24	0.11

**Table 18:** Current amplitude error with externa CT.

	Amplitude Error		
Harmonic Order	Min	Max	Average
II	0.57%	1.6%	1.1%
III	0.49%	1.7%	0.96%
VI	0.29%	2.0%	1.0%
XI	0.16%	1.4%	0.73%

**Table 19:** Current phase error with externa CT.

	Current Phase Error		
Harmonic Order	Min	Max	Average
II	0.017	0.38	0.25
III	0.019	0.44	0.29
VI	0.065	1.8	0.96
XI	0.98	2.4	1.8
